# Scarlet macaw (*Ara macao*) breeding at the Mimbres archaeological site of Old Town (early AD 1100s) in Southwestern New Mexico

**DOI:** 10.1093/pnasnexus/pgad138

**Published:** 2023-06-13

**Authors:** Cyler Conrad, Kimberly Wurth, Travis Tenner, Benjamin Naes, Steven A LeBlanc, Darrell Creel, Katharine Williams, E Bradley Beacham

**Affiliations:** Environmental Protection and Compliance Division, Environmental Stewardship Group, Los Alamos National Laboratory, Los Alamos, NM 87545, USA; Department of Anthropology, University of New Mexico, Albuquerque, NM 87131, USA; Chemistry Division, Nuclear and Radiochemistry Group, Los Alamos National Laboratory, Los Alamos National Laboratory, Los Alamos, NM 87545, USA; Chemistry Division, Nuclear and Radiochemistry Group, Los Alamos National Laboratory, Los Alamos National Laboratory, Los Alamos, NM 87545, USA; Chemistry Division, Nuclear and Radiochemistry Group, Los Alamos National Laboratory, Los Alamos National Laboratory, Los Alamos, NM 87545, USA; Peabody Museum of Archaeology and Ethnology, Harvard University, Cambridge, MA 02138, USA; Department of Anthropology, University of Texas at Austin, Austin, TX 78712, USA; Department of Anthropology, University of New Mexico, Albuquerque, NM 87131, USA; Epsilon Systems Solution Inc., Las Cruces, NM 88005, USA

**Keywords:** scarlet macaw, *Ara macao*, breeding, husbandry, scanning electron microscope, egg

## Abstract

Examination of avian eggshell at the Old Town archaeological site in Southwestern New Mexico, United States of America, indicates that scarlet macaw (*Ara macao*) breeding occurred during the Classic Mimbres period (early AD 1100s). Current archaeological and archaeogenomic evidence from throughout the American Southwest/Mexican Northwest (SW/NW) suggests that Indigenous people bred scarlet macaws at an unknown location(s) between AD 900 and 1200 and likely again at the northwestern Mexico site of Paquimé post-AD 1275. However, there is a lack of direct evidence for breeding, or the location(s) of scarlet macaw breeding itself, within this area. This research, for the first time, provides evidence of scarlet macaw breeding using scanning electron microscopy of eggshells from Old Town.

**Significance Statement**

Scanning electron microscopy of scarlet macaw eggshells from the Old Town archaeological site in southwestern New Mexico confirms the presence of macaw breeding during the early AD 1100s. This record now confirms the presence of a distinct breeding locality for scarlet macaws in the American Southwest prior to, and earlier than, the possible record of macaw breeding at Paquimé in northwestern Mexico post-AD 1275.

## Introduction

An unanswered question pertaining to the husbandry and management of scarlet macaws (*Ara macao*) in the precontact American Southwest/Mexican Northwest (SW/NW) is the degree to which these birds were bred—and more specifically where and when they were bred. Genetic, skeletal, isotopic, and contextual evidence strongly suggests the presence of scarlet macaw breeding at the northwestern Mexico site of Paquimé post-AD 1275, but this site still lacks clear and direct evidence for macaw breeding (1–3). There is also no clearly documented evidence for macaw breeding further north of this area (3–5). For example, past Indigenous people transported scarlet macaws to Chaco Canyon in the AD 1000–1100 range, but there is no evidence for confirmed scarlet macaw breeding (6). This perplexing pattern exists throughout the SW and is especially relevant given that scarlet macaws (or objects created from macaw feathers) appear in archaeological contexts over a wide regional area and long-term period (see Crown and Fladeboe and Taylor (4, 5)). Thus, identifying where scarlet macaw breeding occurred within the SW/NW, and at what time, is crucial for examining the processes that led to the development of human–macaw interaction and management.

**Fig. 1.**
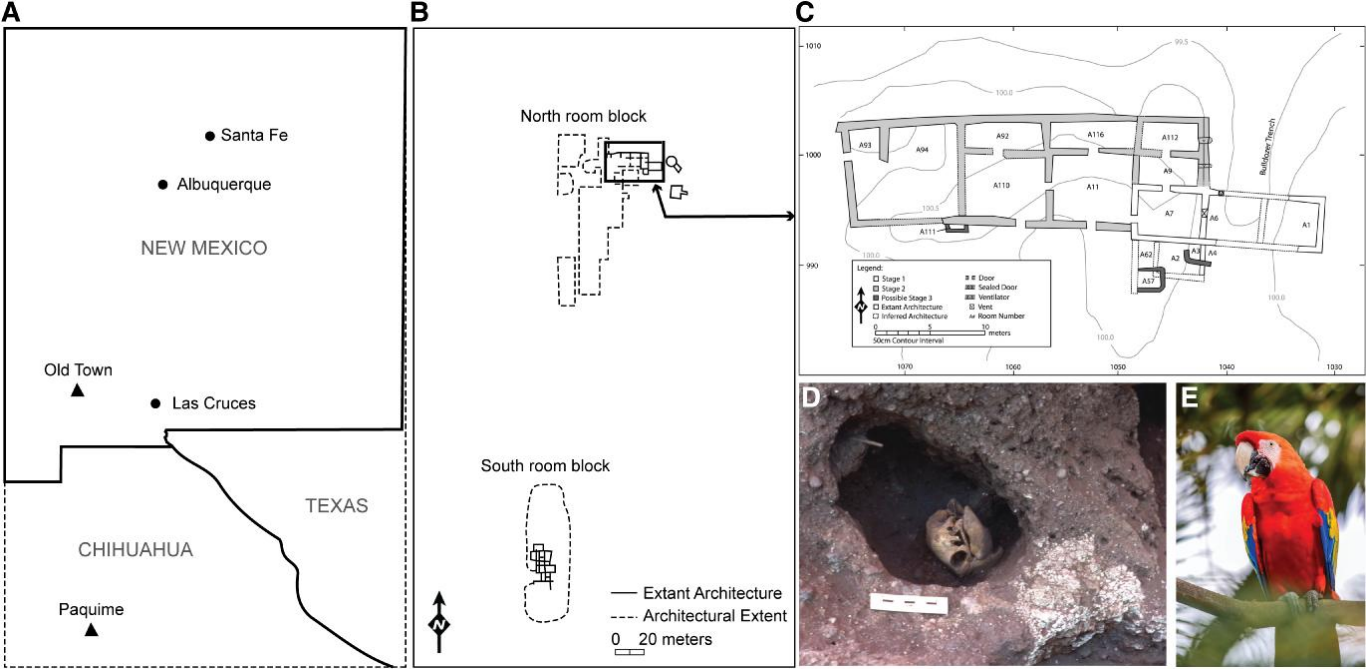
A) The location of Old Town in Southwestern New Mexico and Paquimé in northwestern Mexico; B) a site map of the northern and southern room blocks at Old Town; C) a magnified view of area A; D) room A3: this room had a reinforced wall and hardened floor and included a scarlet macaw burial; and E) finally, a photograph of a modern scarlet macaw.

Here, our research focuses on investigating the identification and ontogenetic age for a sample of avian eggshell recovered from Old Town (LA 1113), a Late Pithouse to Classic Mimbres (AD 750–1150) archaeological site in Southwestern New Mexico, United States of America (7, 8). Excavations at Old Town recovered 14 eggshell specimens that appeared to represent one or more unlaid eggs together among the articulated bones of a breeding-age scarlet macaw burial (osteological identification confirmed by ancient DNA; 9) dating to the early ∼AD 1100s as indicated by context and radiocarbon dating (Fig. 1; 10). Based on results from scanning electron microscopy (SEM), these eggshells exhibit at least two or more ontogenetic ages present within the samples, indicative of multiple eggs. These results confirm that macaw breeding occurred in the SW/NW at Old Town during the Classic Period prior to the AD 1200s.

## Results

All six eggshell samples imaged from Old Town represent macaws (*Ara* sp.; see supplementary material 11 and also Fig. S3). Given the scarlet macaw burial (see George et al. (9)) associated with these fragments and given the tight concentration of eggshell fragments within the body cavity of the macaw, we are confident that the eggshells are from this species but acknowledge that it is currently not possible to distinguish species of macaw from SEM imaging alone (Fig. S2).

**Fig. 2.**
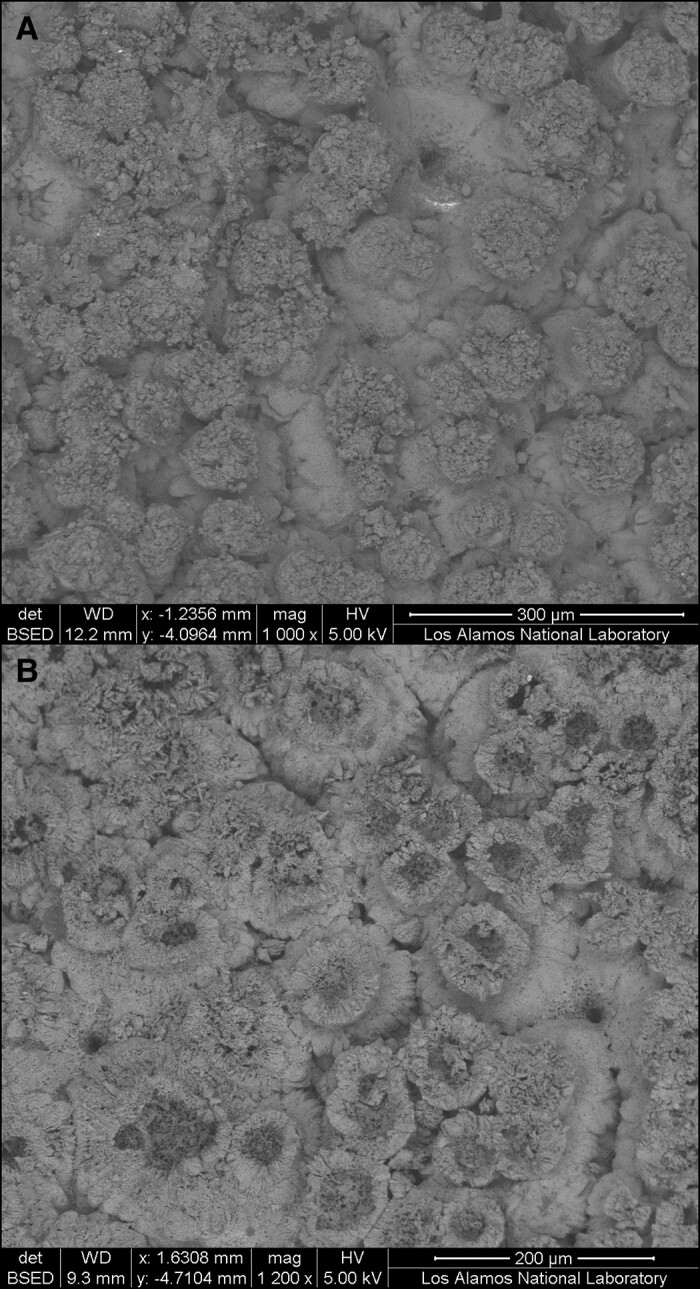
SEM images of the inner surface of two scarlet macaw (*Ara macao*) eggshells from Old Town. A) (#1113.1): little to no reabsorption, and B) (#1113.2): reabsorption present.

In five imaged specimens, there was clear and variable evidence for reabsorption of the mammillary cone with only one specimen lacking evidence for any degree of reabsorption (Figs. 2 and S4–S9)—although this specimen may be a false positive and represent a fragment from the eggshell air sac (see Conrad et al. and Douglass et al. (11, 12)). Each of the five eggshells with evidence for embryotic development (i.e. reabsorption) differed slightly in their ontogenetic age (12). These results indicate that (i) the eggshell specimens from room A3 are from macaws, undoubtedly scarlet macaws, and (ii) at least two or more eggs likely occur in this context (see Supplementary material).

## Discussion

Although numerous archaeological sites dating to the purported era of macaw breeding in the SW/NW (∼AD 900–1200) and at Paquimé (post-AD 1275) include evidence for scarlet macaw skeletal remains, feathers, or iconography, there was, until now, a lack of direct evidence for the occurrence of breeding. Analysis of eggshells associated with a scarlet macaw burial at Old Town indicates that scarlet macaw breeding occurred in the SW/NW during the early AD 1100s. These results significantly shift our understanding of precontact human–macaw interaction and management within the SW/NW because they suggest that there was at least one location with scarlet macaw breeding outside of Mesoamerica during the AD 900s–1200s and that Paquimé was not the sole breeding center and distribution locality for the entire SW/NW region post-AD 1275—if in fact breeding occurred there at all.

Our evidence supports scarlet macaw breeding at Old Town, and future work may help clarify whether this area of the SW/NW also functioned as a separate and distinct breeding center (based on George et al. (9)) that provided a founder population to the broader SW/NW (see also Gilman (13)). With redating of the Mitchell site macaw, all of the scarlet macaws in the Mimbres region date to ∼AD 1000–1130 (Pers. comm. R.J. George and D.J. Kennett, 2023 January 22), which approximately matches the earliest evidence for scarlet macaws in the Ancestral Pueblo region as well. Old Town also has evidence for potential scarlet macaw pens, which would support its role as a possible breeding center. In southern Arizona, scarlet macaws occur in Hohokam contexts dating to AD 600 or 700 (14) which also suggests that while macaw breeding occurred in the SW at Old Town, there may still be earlier breeding locations yet to be identified.

Future analysis of eggshell remains from scarlet macaw contexts and/or archaeological sites with evidence for human–macaw interaction will undoubtedly clarify the extent to which Indigenous people in the precontact SW/NW translocated, bred, and interacted with scarlet macaws originating from a single wild population historically located within Mexico and Central America.

## Materials and methods

Excavations at Old Town produced several contexts with eggshell fragments. Our analysis focused on a collection of eggshells discovered in association with a scarlet macaw burial in area A, room 3 (feature A3–9) at the village (Lot# 994; ARC# 63614). This context contained 14 total fragments and dates to the Late Classic Period (AD 1100s).

We measured the eggshell thickness (in millimeters) of each fragment in triplicate (see data in Conrad et al. (11)) for 10 specimens using Mitutoyo outside point jaw digital calipers—four eggshells were too small to safely measure without potential damage to the sample.

Based on eggshell sample size and the potential presence of an organic membrane, which obscures the mammillary cone surface when present (Fig. S1), we then selected six (*n* = 6/10 measured specimens) fragments for imaging using SEM. To limit potential damage to the shells, we did not use any conductive coating and simply mounted each sample on a Ted Pella SEMClip 18-mm pin mount held in place with a copper clip. We characterized these six samples using a FEI Quanta 200F field emission SEM. Images were collected with an accelerating voltage between 5 and 10 kV, and an FEI spot size setting of 3. We collected secondary electron (SE) and backscatter electron (BSE) images for respective textural and Z-contrast information. However, severe charging occurred on most samples due to lack of a carbon coating; as such, SE images could only be collected for sample 1113.1 (see Conrad et al. (11) which includes supplemental SEM eggshells images not included in the text).

## Supplementary Material

pgad138_Supplementary_Data

## Data Availability

The data associated with this manuscript include a supplemental document with SEM images and associated information. We curated these files at osf.io (see Conrad et al. (11)).
