# Supplementary material for: Scarlet macaw (Ara macao) breeding at the Mimbres archaeological site of Old Town (early AD 1100s) in Southwestern New Mexico
Source: PNAS Nexus. 2023 Jun 13;2(6):pgad138. doi: 10.1093/pnasnexus/pgad138 (PMC10263259; doi:10.1093/pnasnexus/pgad138)
Supplement: pgad138_Supplementary_Data [file pgad138_supplementary_data.zip › PNASNEXUS-PNASNEXUS-2022-01243-T-s01.docx]

**Supplemental Information**

***Eggshell Morphology and Scanning Electron Microscopy***

Analysis of avian eggshell using scanning electron microscopy began in the ~1970s when archaeologists recognized that inner-eggshell mammillary cone morphology differs between taxa (1-4). The outer eggshell crystalline layer protects the mammillary cone structures within, and these cones articulate with an organic membrane layer (see Figure S1) that separates the mammillary cones from the embryo (see figures in 1-4). In general, as the avian embryo develops during its incubation cycle, secreted acids break down the mineral structures of the mammillary cones over time and the essential elements comprising the cone are reabsorbed into the embryo (e.g., calcium for bone development; see 5 for additional details). Various studies confirm that not only does the mammillary cone structure differ between taxa (1-4), but that the morphology of the cone changes during incubation (6). In a controlled turkey (*Meleagris gallopavo*) incubation study, Beacham and Durand (6) identified that reabsorption of the mammillary cone occurs throughout the incubation cycle, and it is possible to identify the period of (or approximate day) of embryotic development based on the visible reabsorption of the mammillary cone (see also 7 – known as aging the eggshell or determining the “ontogenetic age” of the eggshell). In this study, we utilized the same approach: 1) imaging the eggshells to determine their taxonomic identification based on mammillary cone morphology, and 2) examining the reabsorption patterns present in each shell sample to determine their degree of incubation or ontogenetic age.

Within the American Southwest/Northwest Mexico and Mesoamerica, previous eggshell analyses have largely focused on the identification and aging of turkey eggs (6-9). However, turkeys were not the only bird managed by Indigenous peoples within this region, and eggshells recovered from archaeological sites may represent other avian taxa (e.g., macaws [*Ara* sp.]). Modern military macaw (*Ara militaris*) eggshell images published (6) and available for study help clearly distinguish between these two avian taxa (Figures S2 and S3). In macaw eggshells, the elongated crystal palisade structure supporting the mammillary cone is roughly circular in shape with diagnostic fluted edges. The overall shape of the structure looks surprisingly similar to a traditional bundt cake pan. This is in sharp contrast to turkey eggs, as a comparison, which Sidell (4) describes as typically elliptical or oval. Turkey egg palisade structures have more defined and ‘straight’ or angular edges. These images and a review of published scanning electron microscopy images for a variety of avian eggshells confirm that the Old Town eggshells represent macaws.

While all of the turkey eggshell analyses to date involve qualitative 2-dimensional imaging and examination, recently published research on common ostrich (*Struthio camelus*) and cassowary (Casuariidae) eggshell provides a new, quantitative, 3-dimensional approach (10-11). This research, by Douglass and her colleagues, is groundbreaking in its ability to create a standardized, reproducible technique for eggshell studies. While our work still focused on a 2D approach, we use a similar “3-stage” classification system to identify the degree of eggshell reabsorption in the Old Town macaw eggs. A holistic analysis of the 3-stage model involves a quantitative, statistical analysis using 3D approaches (e.g., micro-computed tomography; see 11), but here we classify our eggs based on a qualitative examination of the eggshell morphology. The 3-stage classification (11) includes:

Early stage – minimal to no reabsorption of the mammillary cones

Middle stage – pitting and reabsorption present (or beginning) in the mammillary cones

Late stage – significant pitting and reabsorption throughout all mammillary cones

Based on images present for each eggshell sample from Old Town, all three stages are present. In specimen #1113.1 (Figure S4) there is minimal to no reabsorption present (“early stage”), but it is possible that this fragment represents a false positive signal since the air sac (or air cell) in an eggshell prevents reabsorption throughout the incubation cycle (see 11 and 6).

Three eggshells (#1113.5, #1113.7, #1113.8) clearly fall into the “middle stage” since they have visible evidence of reabsorption for certain mammillary cones, and intact cones without reabsorption for others (Figures S5-S7).

Finally, two eggshell specimens (#1113.2 and #1113.4) fall into the “late stage” since they exhibit evidence for significant pitting and reabsorption throughout most to all of the mammillary cones present (Figures S8-S9).

***A Note on the Old Town Macaws***

Skeletal remains of at least two other adult macaws dating to the early AD 1100s were also recovered at Old Town, one identified as a probable scarlet macaw on the basis of osteological traits, the other identifiable only to *Ara* sp. Though sex is unknown for these individuals, it is plausible a breeding pair existed at Old Town. These few macaw remains derive from a relatively small excavation sample from a very large Classic period pueblo, and it is entirely possible that additional macaws were present there at that time. In addition, small reinforced masonry enclosures of an appropriate size for housing macaws were excavated at Old Town in the area where the macaw burial and other macaw bones were found, Room A3 being one (12). There was no archaeological evidence from this context to suggest a disturbance to the macaw skeleton or eggshell remains.

**References**

1. C. Keepax, Identification of Avian Egg Shell from archaeological sites and the potential use of the Scanning electron Microscope, (SEM) (Ancient Monuments Laboratory Report, 1977).
2. C.A. Keepax, Avian Egg-shell from Archaeological Sites. *J. of Arch. Sci.* 8, 315-355 (1981).
3. E.J. Sidell, A Methodology for the Identification of Avian Eggshell from Archaeological Sites. *Archaeofauna* 2, 45-51 (1993).
4. E.J. Sidell, A Methodology for the Identification of Archaeological Eggshell (MASCA, The University Museum of Archaeology and Anthropology, University of Pennsylvania, 1993).
5. A.L. Romanoff, A.J. Romanoff, The Avian Egg (Wiley, 1949).
6. E.B. Beacham, S.R. Durand, Eggshell and the archaeological record: new insights into turkey husbandry in the American Southwest. *J. of Arch. Sci.* 34, 1610-1621 (2007).
7. C. Conrad, E.L. Jones, S.D. Newsome, D.W. Schwartz, Bone isotopes, eggshell, and turkey husbandry at Arroyo Hondo Pueblo. *J. of Arch. Sci. Rep.* 10, 566-574 (2016).
8. C. Conrad, Eggshells and Gastroliths from Room 28: Turkey Husbandry at Pueblo Bonito, in P.L. Crown (ed.) *The House of the Cylinder Jars: Room 28 in Pueblo Bonito, Chaco Canyon* (University of New Mexico Press, 2020).
9. H.A. Lapham, G.M. Feinman, L.M. Nicholas, Turkey husbandry and use in Oaxaca, Mexico: A contextual study of turkey remains and SEM analysis of eggshell from the Mitla Fortress. *J. of Arch. Sci. Rep.* 10, 534-546 (2016).
10. Douglass, K., D. Gaffney, T.J. Feo, P. Bulathsinhala, A.L. Mack, M. Spitzer, G.R. Summerhayes, Late Pleistocene/Early Holocene sites in the montane forests of New Guinea yield early record of cassowary hunting and egg harvesting. *PNAS* 118(40), e2100117118 (2021).
11. Douglass, K., P. Bulathsinhala, T.J. Feo, T. Tighe, S. Whittaker, Z. Brand, H. James, T. Rick, Modeling avian eggshell microstructure to predict ontogenetic age and reveal patterns of human-avifauna interaction. *J. of Arch. Sci.* 133, 105442 (2021).
12. D. Creel, M. Cannon, J. Broughton, C. Francis, The Significance of Birds at Old Town and other Mimbres Sites. *Proceedings of the 18^th^ Mogollon Conference*. (Friends of Mogollon Archaeology, Las Cruces, NM, 2015).

**Supplemental Figures**


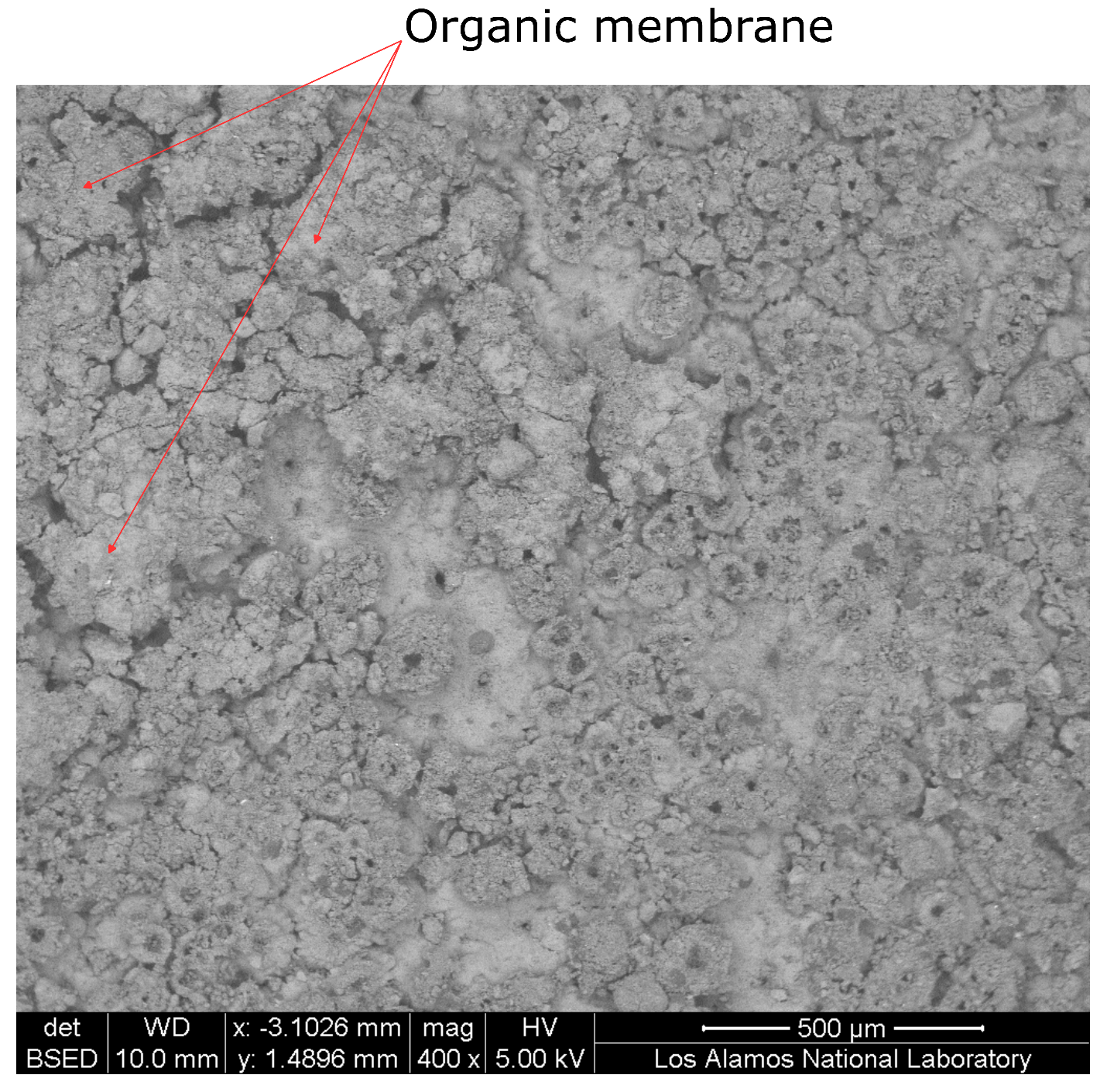


Figure S1. An eggshell fragment from Old Town (#1113.4) with fragments of the organic membrane present.


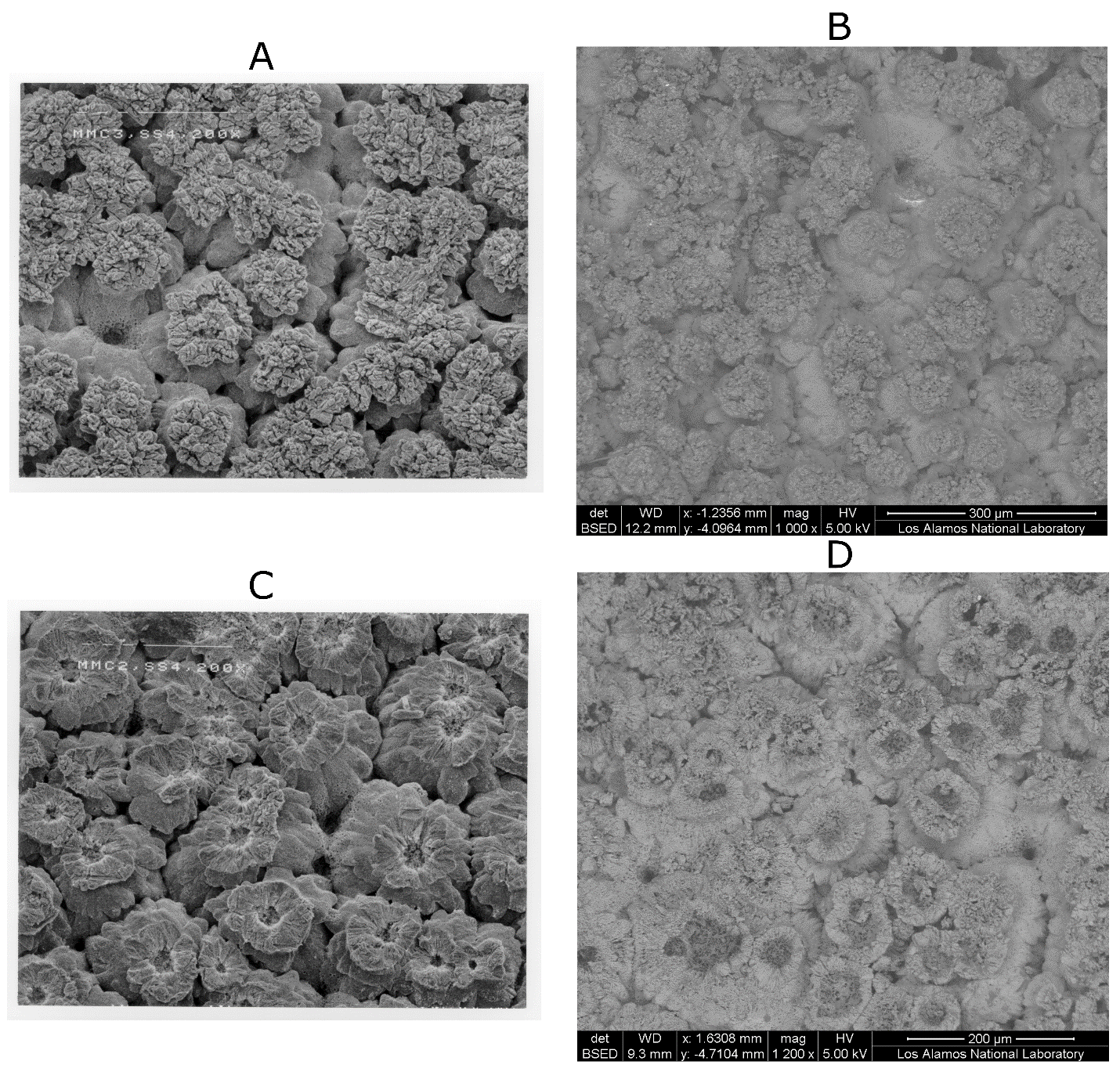


Figure S2. A comparison of modern military macaw (*Ara militaris*) eggshells published by Beacham and Durand (6) and archaeological eggshells from Old Town: A) Military macaw eggshell with no reabsorption, B) Old Town macaw eggshell with no reabsorption (#1113.1), C) Military macaw eggshell with reabsorption, and D) Old Town macaw eggshell with reabsorption (#1113.2).


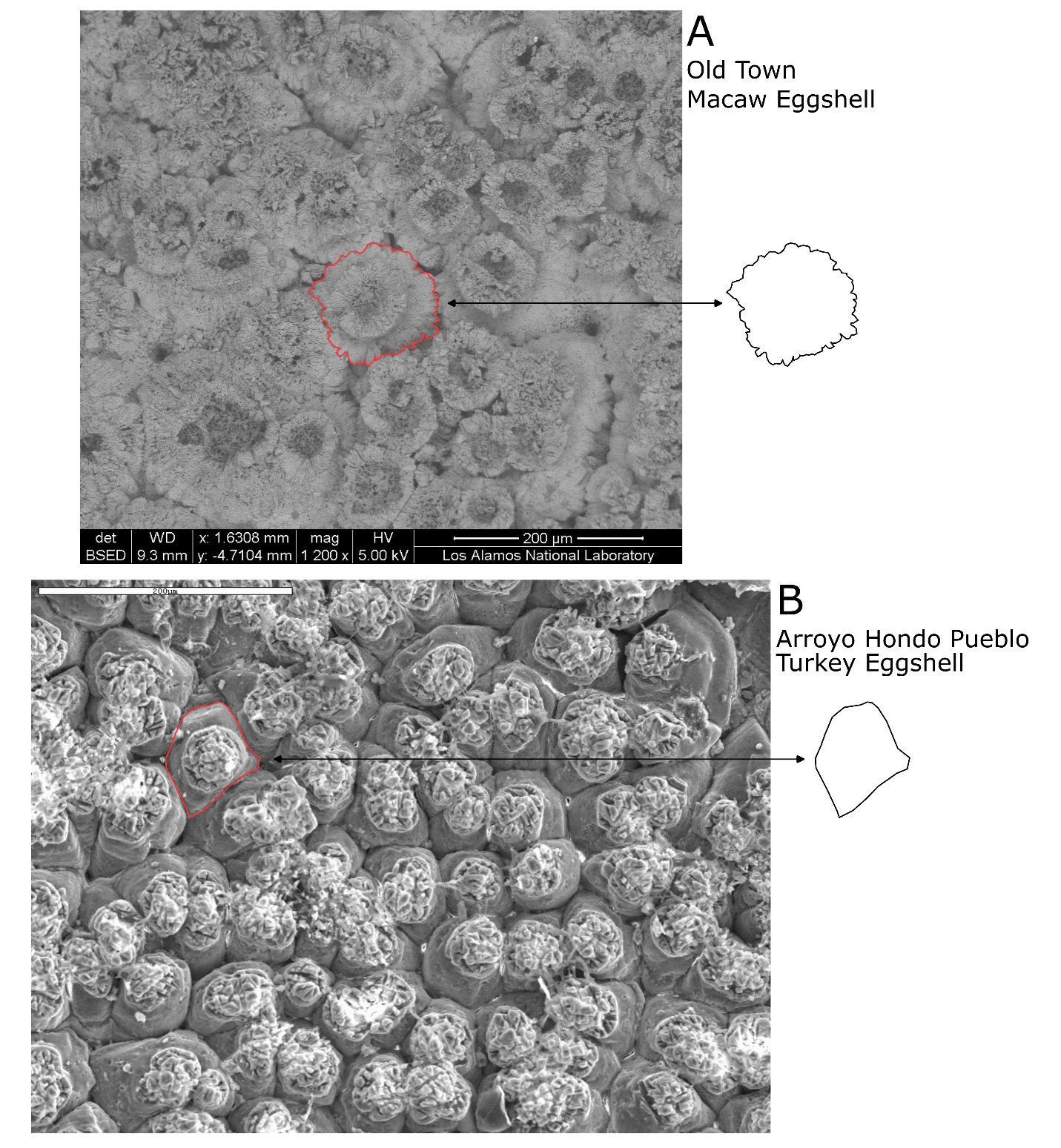


Figure S3. A comparison of the crystal palisade structure supporting the mammillary cone in a macaw eggshell (#1113.2) from Old Town (A) and a turkey eggshell from Arroyo Hondo Pueblo (B; see 7).


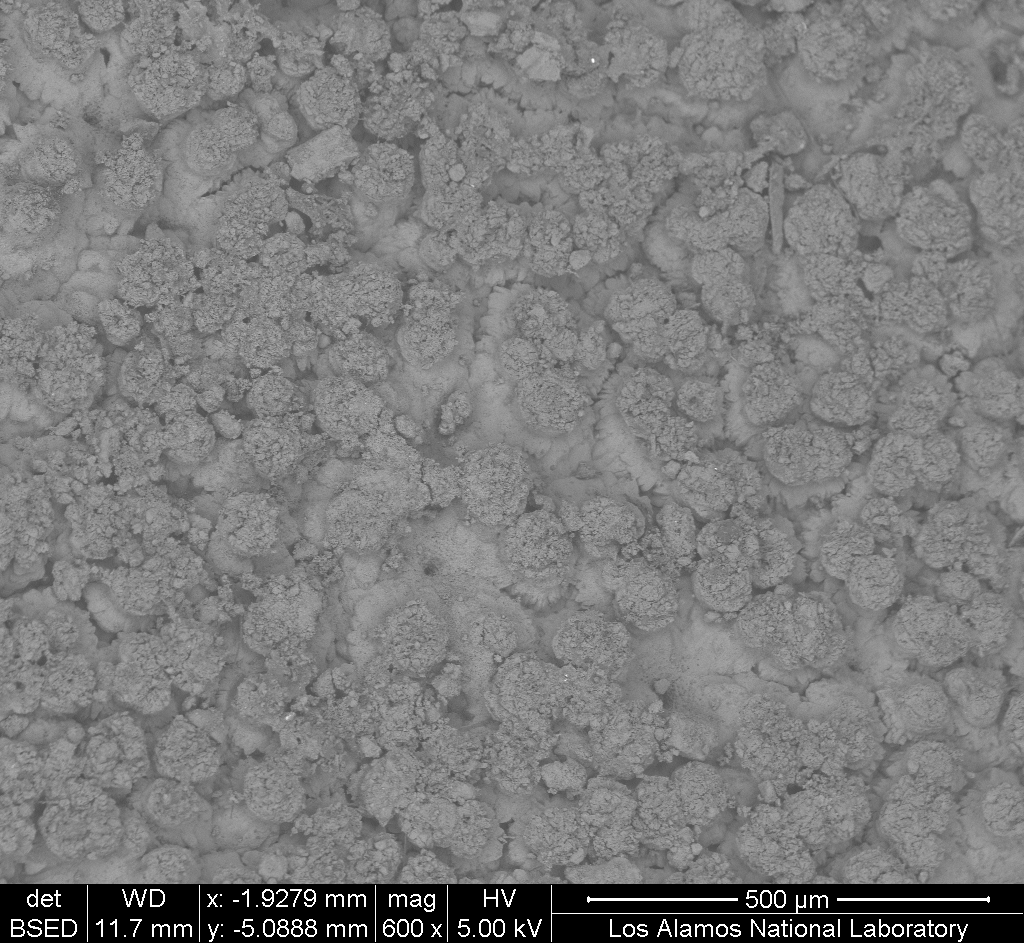
 Figure S4. Old Town macaw eggshell sample 1113.1.


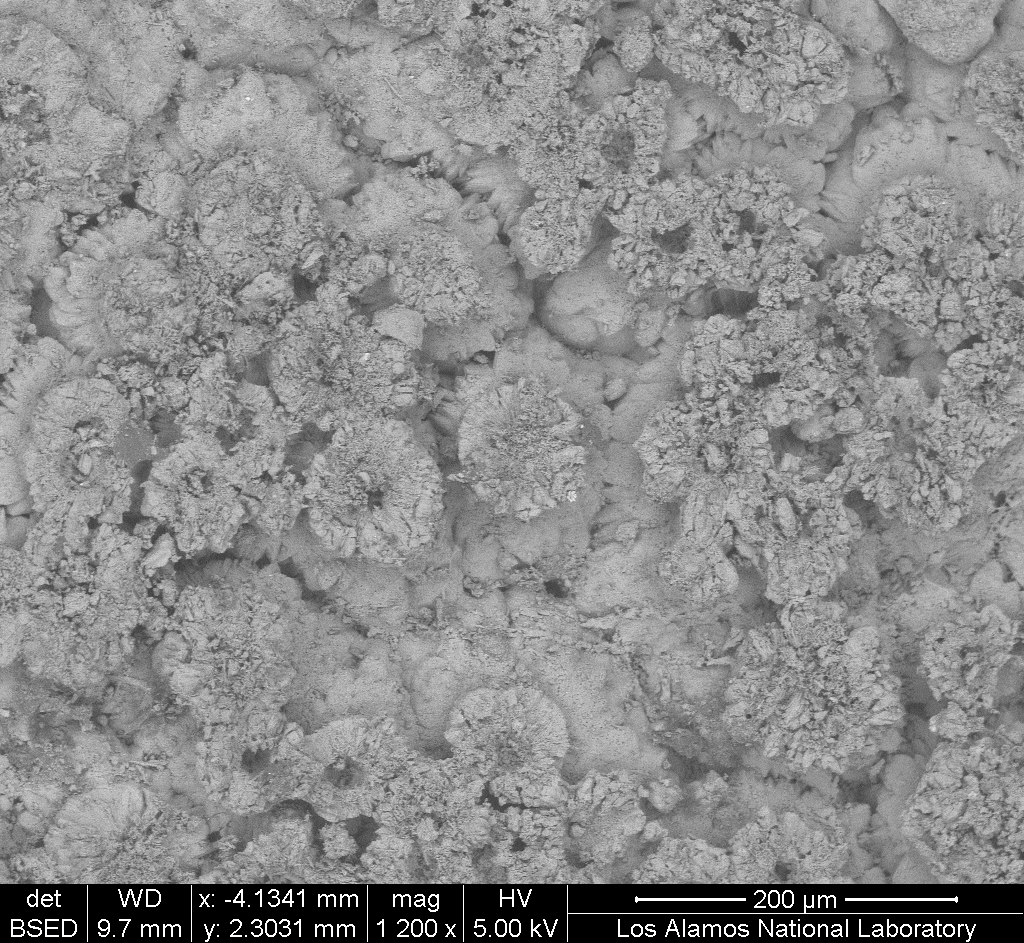
 Figure S5. Old Town macaw eggshell sample 1113.5.


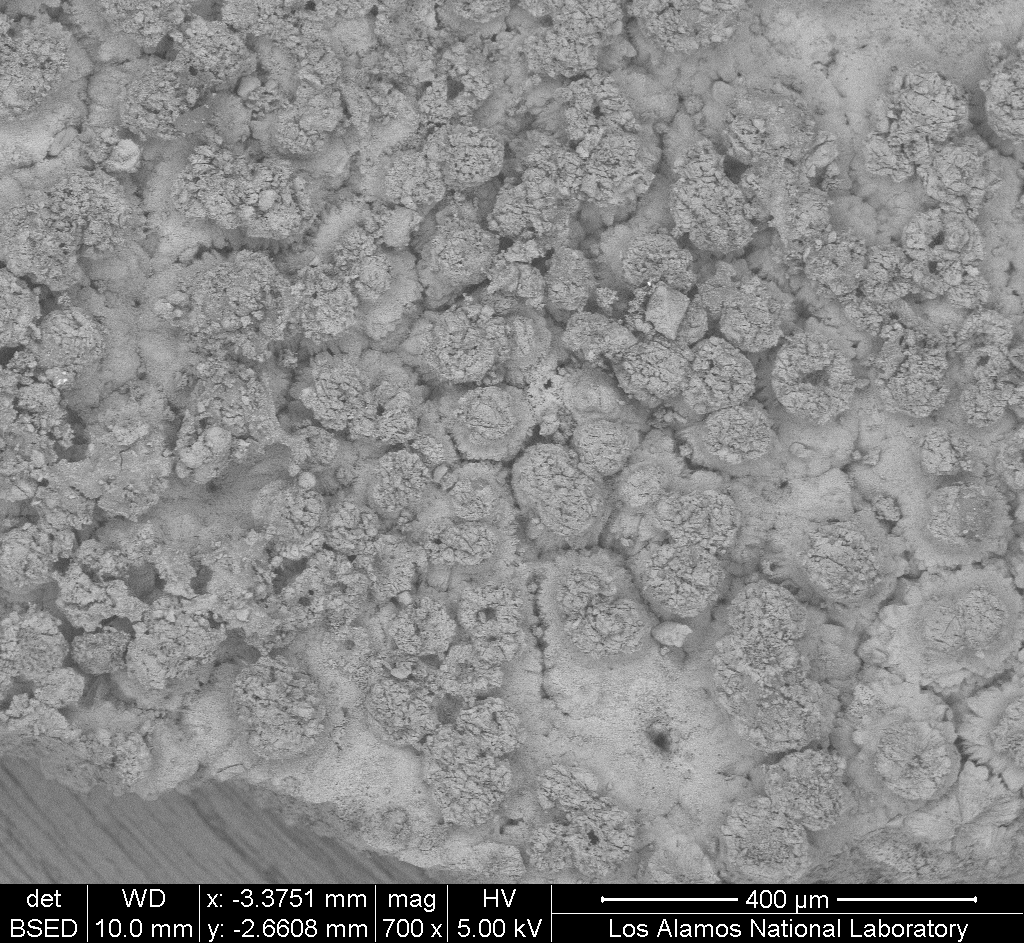
 Figure S6. Old Town macaw eggshell sample 1113.7.


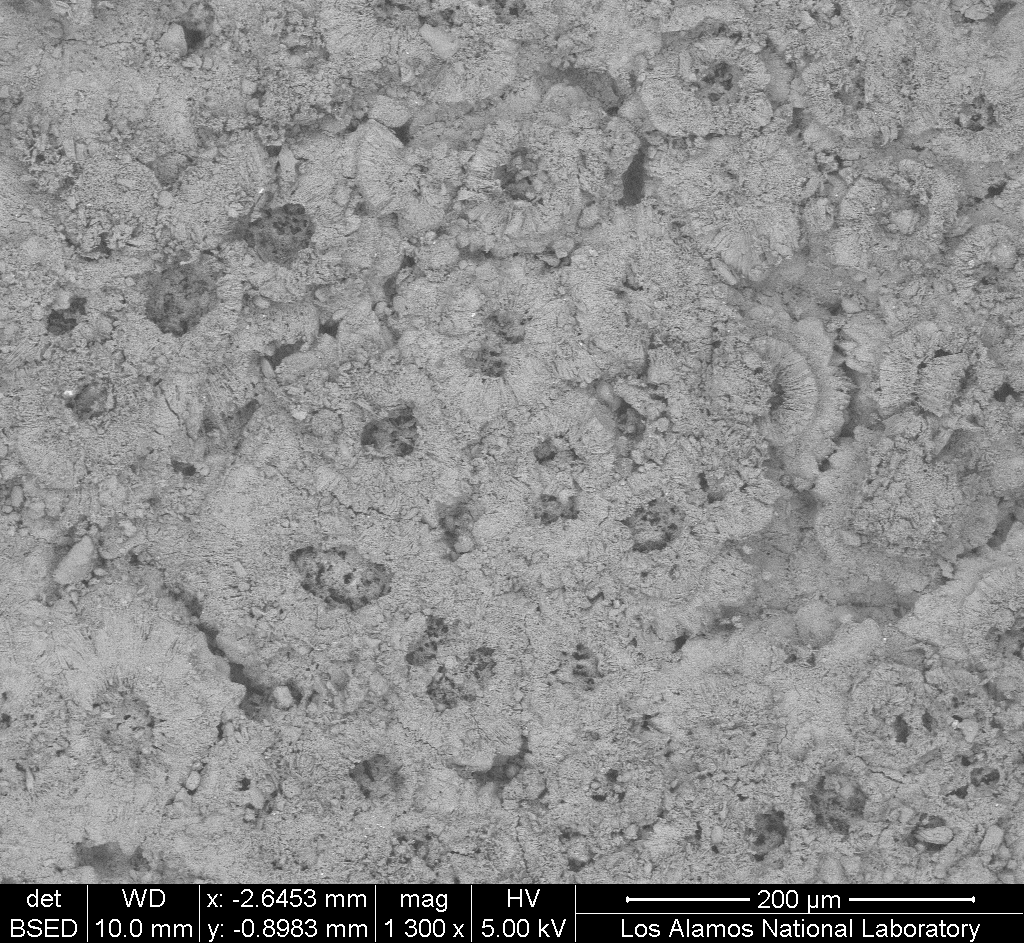
 Figure S7. Old Town macaw eggshell sample 1113.8.


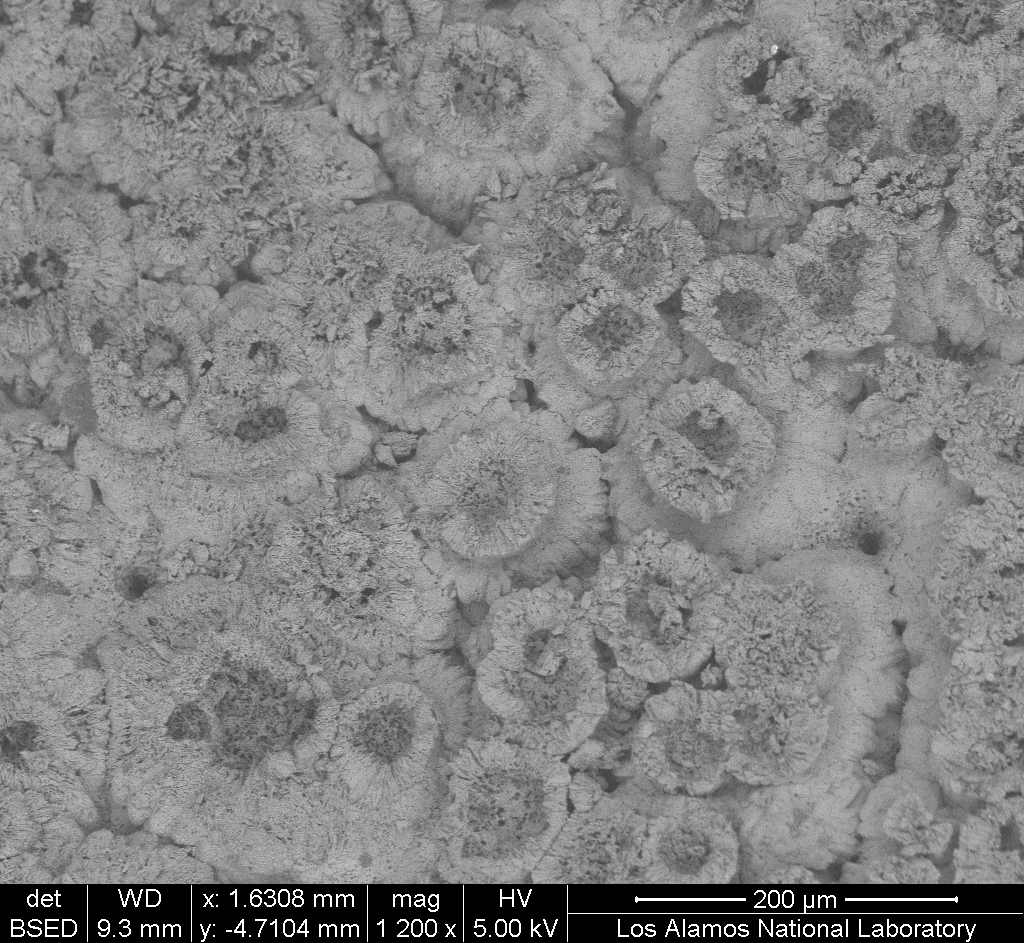
 Figure S8. Old Town macaw eggshell sample 1113.2.


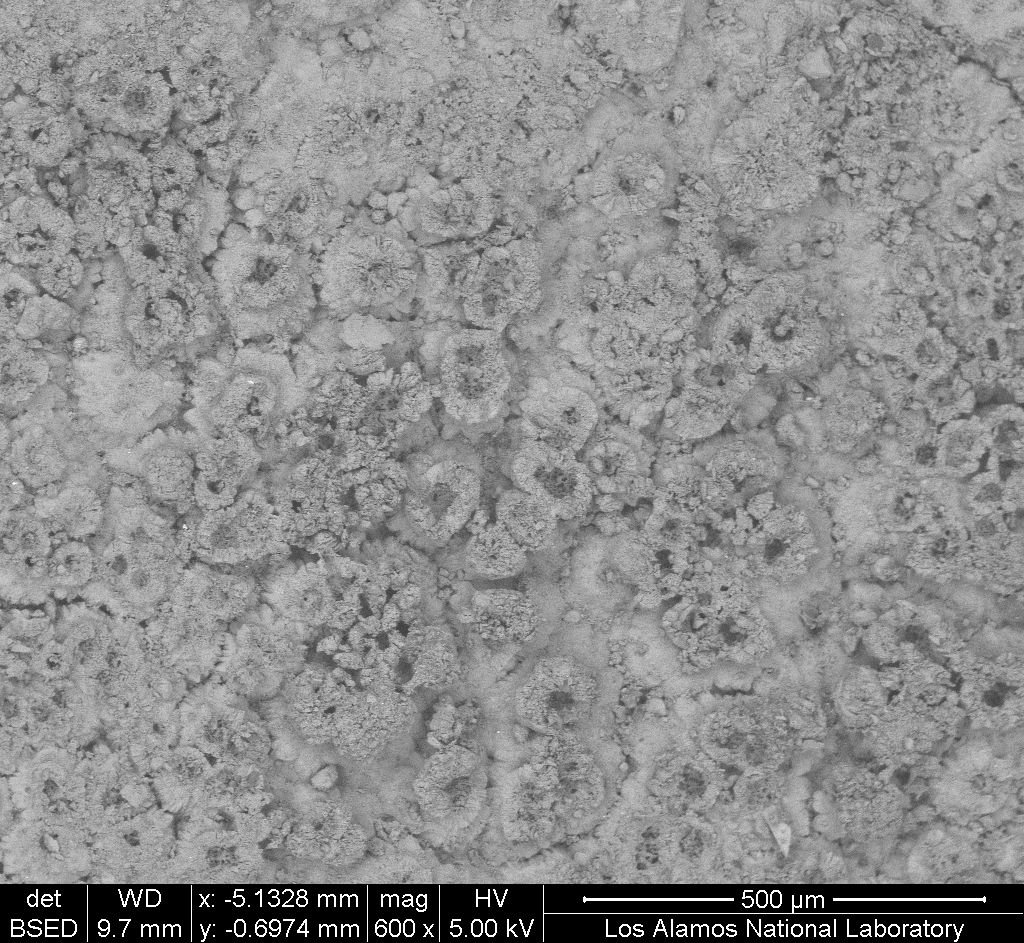
 Figure S9. Old Town macaw eggshell sample 1113.4.
